# New from Old: Thorectandrin Alkaloids in a Southern Australian Marine Sponge, *Thorectandra choanoides* (CMB-01889)

**DOI:** 10.3390/md19020097

**Published:** 2021-02-09

**Authors:** Shamsunnahar Khushi, Angela A. Salim, Ahmed H. Elbanna, Laizuman Nahar, Robert J. Capon

**Affiliations:** Institute for Molecular Bioscience, The University of Queensland, St. Lucia, QLD 4072, Australia; s.khushi@imb.uq.edu.au (S.K.); a.salim@uq.edu.au (A.A.S.); a.elbanna@imb.uq.edu.au (A.H.E.); laboni4@yahoo.com (L.N.)

**Keywords:** *Thorectandra choanoides*, tryptophan alkaloid, indoleamine 2,3-dioxygenase, aplysinopsins, GNPS molecular network

## Abstract

*Thorectandra choanoides* (CMB-01889) was prioritized as a source of promising new chemistry from a library of 960 southern Australian marine sponge extracts, using a global natural products social (GNPS) molecular networking approach. The sponge was collected at a depth of 45 m. Chemical fractionation followed by detailed spectroscopic analysis led to the discovery of a new tryptophan-derived alkaloid, thorectandrin A (**1**), with the GNPS cluster revealing a halo of related alkaloids **1a**–**1n**. In considering biosynthetic origins, we propose that *Thorectandra* *choanoides* (CMB-01889) produces four well-known alkaloids, 6-bromo-1′,8-dihydroaplysinopsin (**2**), 6-bromoaplysinopsin (**3**), aplysinopsin (**4**), and 1′,8-dihydroaplysinopsin (**10**), all of which are susceptible to processing by a putative indoleamine 2,3-dioxygenase-*like* (IDO) enzyme to **1a**–**1n**. Where the 1′,8-dihydroalkaloids **2** and **10** are fully transformed to stable ring-opened thorectandrins **1** and **1a**–**1b**, and **1h**–**1j**, respectively, the conjugated precursors **3** and **4** are transformed to highly reactive Michael acceptors that during extraction and handling undergo complete transformation to artifacts **1c**–**1g**, and **1k**–**1n**, respectively. Knowledge of the susceptibility of aplysinopsins as substrates for IDOs, and the relative reactivity of Michael acceptor transformation products, informs our understanding of the pharmaceutical potential of this vintage marine pharmacophore. For example, the cancer tissue specificity of IDOs could be exploited for an immunotherapeutic response, with aplysinopsins transforming in situ to Michael acceptor thorectandrins, which covalently bind and inhibit the enzyme.

## 1. Introduction

Marine sponges of the genus *Thorectandra* are a rich source of structurally diverse metabolites with novel scaffolds. Examples include, the 1988 report of the sesterterpenes manoalide 25-monoacetate and thorectolide 25-monoacetate from *Thorectandra excavates* collected near Darwin, Australia [1]; the 1995 report of a furanoditerpene from a Southern Australian *Thorectandra choanoides* [2]; and the 2001 and 2002 reports of sesterterpenes thorectandoles A–E from a Palauan *Thorectandra* sp. [3,4]. In addition to terpenes, many alkaloids were also reported from *Thorectandra* sp., including β-carboline alkaloids (i.e., thorectandramine [5], 1-deoxysecofascaplysin A, and fascaplysin [6]), tryptophan alkaloids (i.e., 1′,8-dihydroaplysinopsin and (1*H*-indole-3-yl)acetic acid [7]), and brominated tryptophan alkaloids (i.e., 6-bromo-1′,8-dihydroaplysinopsin, 6-bromo-1′-hydroxy-1′,8-dihydro-aplysinopsin, 6-bromo-1′-methoxy-1′,8-dihydroaplysinopsin, (–)-5-bromo-*N,N*-dimethyl-tryptophan, and (+)-5-bromohypaphorine [7]).

**Figure 1:**
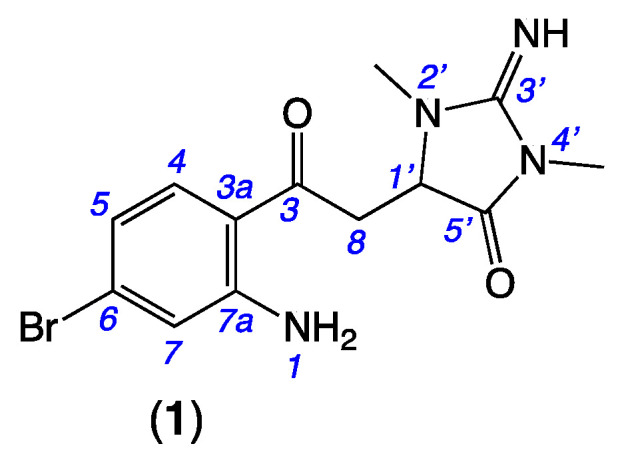
Thorectandrin A (**1**).

Recently, a GNPS molecular networking analysis was employed on 960 Southern Australian marine sponges, to map the chemical space of natural products, which resulted in the isolation of rare indolo-imidazole alkaloids, trachycladindoles H–M [8], new sesterterpene butenolides, cacolides A–L and cacolic acids A–C [9], and new sesquiterpenes, dysidealactams A–F, and dysidealactones A–B [10]. In this report, we present the discovery of a new class of tryptophan-derived alkaloid, thorectandrin A (**1**) (Figure 1), from a Great Australian Bight specimen of *Thorectandra choanoides,* prioritized for chemical investigation, based on GNPS molecular networking analysis of the same library of Southern Australian sponges.

## 2. Results and Discussion

### 2.1. GNPS Molecular Networking to Explore New Chemistry

To search for new marine natural products, 960 *n*-BuOH soluble partitions from the EtOH extracts of a library of Southern Australian marine sponges and 95 authentic standards (previously isolated from marine sponges) from the Capon lab were assembled and subjected to UPLC-QTOF-MS/MS analysis. The resulting data were used to create a consolidated GNPS molecular network (Figure S1). In this molecular network, we found a specific molecular cluster (Figure S2) associated with *Thorectandra choanoides* (CMB-01889) (collected in 1995 during deep water scientific trawling in the Great Australian Bight), which did not co-correlate with any metabolites found in the other 959 sponge extracts, or any of our authentic marine natural products. Following isolation, detailed spectroscopic analysis identified a new alkaloid scaffold, thorectandrin A (**1**) (Figure 1), while mass spectrometry (MS) data revealed molecular formulae for a host of structurally related analogues (**1a**–**1n**) in the same GNPS cluster. Note: All *Thorectandra choanoides* (CMB-01889) chemistry in this report are displayed as free bases, although all were isolated, and where appropriate, characterised as the trifluoroacetic acid salts.

### 2.2. Thorectandrin A (***1***)

**Figure 2:**
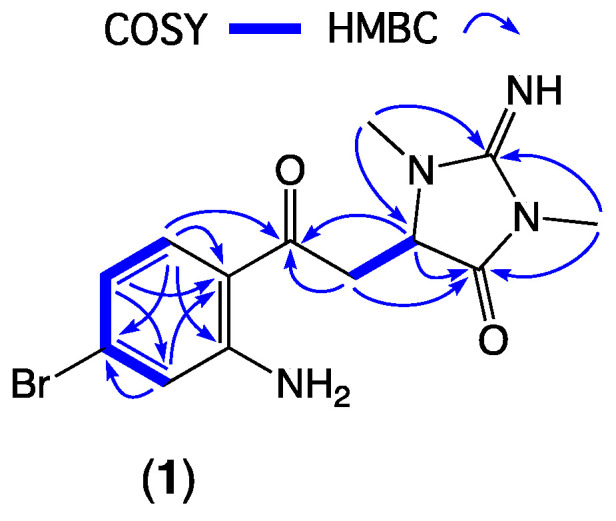
Diagnostic 2D NMR correlations for thorectandrin A (**1**).

HRESI (+) MS analysis of **1** returned a molecular formula (C_13_H_15_BrN_4_O_2_, Δmmu +2.6) incorporating eight double-bond equivalents (DBE). The NMR (methanol-*d*_4_) data for **1** (Table 1, Figures S5–S10) revealed resonances attributed to a ketone (δ_C_ 197.8, C-3), two sp^2^ amido/imino carbonyl carbons (δ_C_ 160.3, C-3′ and 173.8, C-5′), and a 1,2,4-trisubstituted aromatic ring (δ_C_ 154.1, C-7a; 133.9, C-4, 131.1, C-6; 120.7, C-7; 119.4, C-5; 116.1, C-3a; δ_H_ 7.67, d, *J* = 8.7 Hz, H-4; 6.73, br d, *J* = 8.7 Hz, H-5 and 6.97, br s, H-7), accounting for seven DBE, and requiring that **1** incorporate an additional ring system. Further analysis of NMR data revealed resonances for two *N*-methyls (δ_H_ 3.09, s, 2′N-Me and 3.26, s, 4′N-Me) and a deshielded diastereotopic methylene-methine spin system (δ_H_ 3.94, dd, *J* = 19.0 and 3.5 Hz, H-8a; 3.67, dd, *J* = 19.0 and 3.9 Hz, H-8b; and 4.56, dd, *J* = 3.9 and 3.5 Hz, H-1′). HMBC correlations from 2′N-Me and 4′N-Me to a common C-3′ guanidino carbon; from 4′N-Me and H-1′ to a common carbonyl C-5′ and from 2′N-Me to C-1′ (δ_C_ 60.8) suggested the presence of a 2-imino-1,3-dimethylimidazolidin-4-one ring system, similar to that observed in the well-known *Thorectandra* metabolite aplysinopsin [11]. HMBC correlations from H-4, H-8a, H-8b, and H-1′ to C-3 confirmed that the aromatic ring was connected to the imidazolidinone ring, through a common carbon C-3, establishing the structure of thorectandrin A (**1**), as shown (Figure 2). Comparison of 1D and 2D NMR data of **1** with that of the known sponge metabolite 6-bromo-1′,8-dihydroaplysinopsin (**2**) [7] (Figure 2) revealed the main differences as the disappearance of resonances for H-2/C-2 (δ_H_ 7.09, δ_C_ 124.8) in **2** and replacement of the resonance of an sp^2^ carbon C-3 (δ_C_ 106.5) in **2** with an α,β-unsaturated ketone (δ_C_ 197.8) in **1,** consistent with a ring-opened analogue of **2.**

**Scheme 1:**
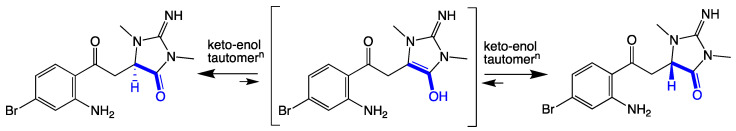
Keto-enol tautomerization of thorectandrin A (**1**).

As thorectandrin A (**1**) did not exhibit a measurable [α]_D_ or ECD spectrum (Figure S12), we propose it exists as a racemate, induced by a slow keto-enol tautomerization during long-term storage (~25 years) in EtOH (Scheme 1). That the proposed racemization is slow was evident when **1** did not incorporate deuterium, when stored in deuterated methanol for several days.

Thorectandrin A (**1**) did not exhibit growth inhibitory activity against the Gram-positive bacterium *Bacillus subtilis* (ATCC 6051), the Gram-negative *Escherichia coli* (ATCC 11775), the fungus *Candida albicans* (ATCC 10231), or human colorectal (SW620) and lung (NCI-H460) carcinoma cells, at concentrations up to 30 μM (Figures S13 and S14).

### 2.3. Plausible Biosynthetic Pathway

**Scheme 2:**
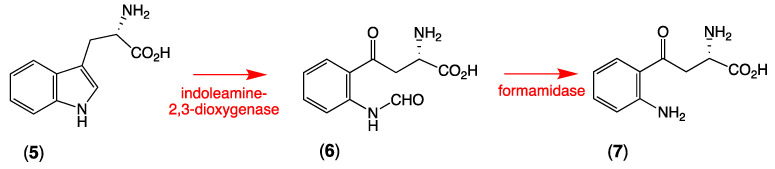
Biosynthetic conversion of L-tryptophan (**5**) to *N*-formyl-L-kynurenine (**6**) to L-kynurenine (**7**).

Aplysinopsins are tryptophan-derived marine natural products, which were isolated from many genera of sponges and scleractinian corals, as well as from one sea anemone and one nudibranch [11], the latter most likely a dietary input from nudibranches feeding on sponges. Typical exemplars include 6-bromoaplysinopsin (**3**) and aplysinopsin (**4**). In turning our attention to the likely biosynthesis of thorectandrin A (**1**), we considered the metabolic relationship between L-tryptophan (**5**), *N*-formyl-L-kynurenine (**6**), and L-kynurenine (**7**), and the fact that indoleamine 2,3-dioxygenase is known to convert **5** to **6**, which undergoes facile hydrolysis by a formamidase to **7** (Scheme 2) [12].

**Scheme 3:**
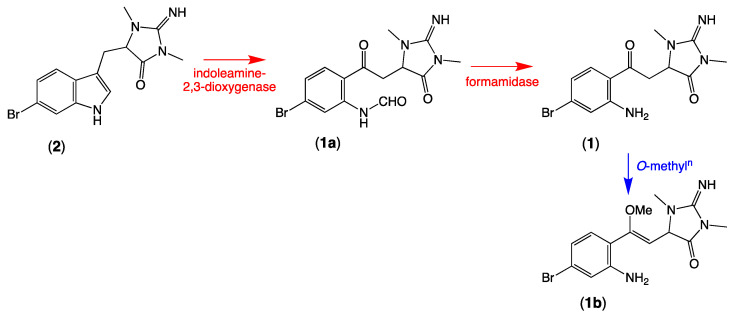
Possible biosynthetic link between 6-bromo-1′,8-dihydroaplysinopsin (**2**), *N*-formyl thorectandrin A (**1a**), *O*-methylthorectandrin A (**1b**), and thorectandrin A (**1**).

Inspired by this sequence of transformations, we hypothesised that a comparable indoleamine 2,3-dioxygenase-*like* enzyme in *Thorectandra choanoides* (CMB-01889) converts 6-bromo-1′,8-dihydroaplysinopsin (**2**) to its ring-opened *N*-formyl derivative (**1a**), which is then rapidly hydrolyzed to thorectandrin A (**1**) (Scheme 3). Although **2** is reported as a sponge natural product [7], its absolute configuration (even enantiopurity) remains unassigned. Based on our experience, a possible challenge to assigning an absolute configuration to **2** might be enantiopurity, due to a propensity for keto-enol mediated epimerisation/racemisation.

**Scheme 4:**
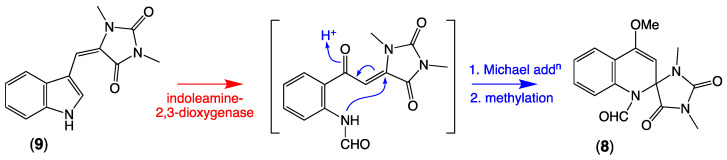
Possible biosynthetic link between (*Z*)-3′-deimino-3′-oxoaplysinopsin (**9**) and spiroreticulatine (**8**).

Armed with knowledge of the new thorectandrin scaffold and its likely biosynthetic relationship to the vintage aplysinopsin scaffold, we turned our attention to the literature and noted a 2015 report of racemic spiroreticulatine (**8**) from the South China Sea marine sponge *Fascaplysinopsis reticulata* [13], and a subsequent 2019 report from the same source of the known sponge alkaloid (*Z*)-3′-deimino-3′-oxoaplysinopsin (**9**), as a co-metabolite with **1b** [14]. Although **8** was initially ascribed a plausible biosynthesis involving condensation of indole-3-carboxaldehyde and 1,3-dimethylhydantoin, this hypothesis seems highly improbable. A far more likely pathway would see ring opening of the indole heterocycle in the cometabolite **9**, delivering a reactive Michael acceptor intermediate that undergoes non-stereoselective (enzyme or non-enzyme mediated) intramolecular Michael addition to racemic **8** (see Scheme 4).

### 2.4. Other Thorectandrin Co-Metabolites

**Figure 3:**
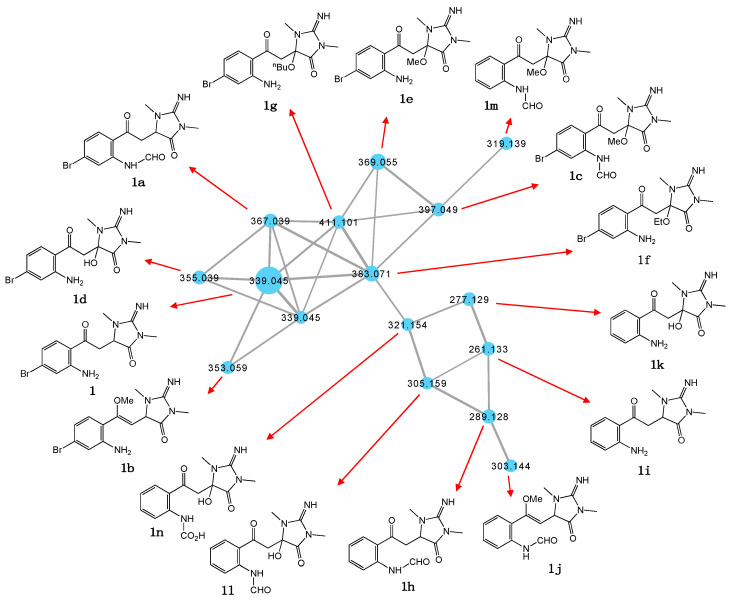
Structures attributed to nodes in the thorectandrin GNPS cluster.

While the thorectandrin GNPS cluster revealed a number of related metabolites, due to low abundance and a lack of sponge biomass, it was not possible to isolate and acquire definitive spectroscopic data to secure unambiguous structure assignments. Notwithstanding, we did acquire molecular formulae for many minor compounds, and on the basis of these measurements and biosynthetic considerations we tentatively propose structures, as shown in Table 2 and Figure 3. For example, based on differences in MW and elemental composition with **1**, we detected a node attributed to the hypothesized *N*-formyl **1a** (M + H *m*/*z* 367, C_14_H_15_BrN_4_O_3_) and *O*-methyl **1b** (M + H *m*/*z* 353, C_14_H_17_BrN_4_O_2_) (Scheme 3).

**Scheme 5:**
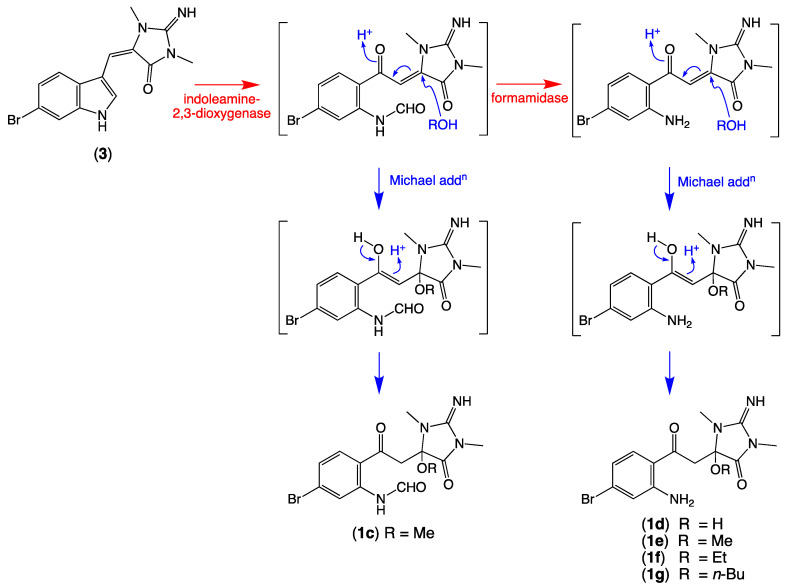
Possible biosynthetic link between 6-bromoaplysinopsin (**3**) and the solvolysis artifacts **1c**–**1g.**

We also detected nodes that were *tentatively* attributed to a methanol (**1c**, M + H *m*/*z* 397, C_15_H_17_BrN_4_O_4_) adduct of **1a**, and water (**1d**, M + H *m*/*z* 355, C_13_H_15_BrN_4_O_3_), methanol **(1e,** M + H *m*/*z* 369, C_14_H_17_BrN_4_O_3_), ethanol (**1f**, M + H *m*/*z* 383, C_15_H_19_BrN_4_O_3_), and *n*-butanol (**1g**, M + H *m*/*z* 411, C_17_H_23_BrN_4_O_3_) adducts of **1** (Table 2, Figure 3). While these adducts **1c**–**1g** are believed to be solvolysis artifacts induced by long-term storage of *Thorectandra choanoides* (CMB-01889) in aqueous ethanol, followed by *n*-butanol partitioning and the use of methanol to dissolve dried extract, the absence of a rational solvolysis pathway from **1a** to **1c**, and **1** to **1d**–**1g**, warranted consideration. We hypothesized that in addition to 6-bromo-1′,8-dihydroaplysinopsin (**2**), *Thorectandra choanoides* (CMB-01889) produces 6-bromoaplysinopsin (**3**), which was comparably transformed by an indoleamine 2,3-dioxygenase-*like* enzyme to reactive Michael adducts (*N*-formyl-Δ^1′−8^-thorectandrin A and Δ^1′−8^-thorectandrin A), with both undergoing Michael addition solvolysis during storage, fractionation and handling, to **1c**–**1g** (Scheme 5). A recent review highlights the prevalence of solvolysis adduct artifacts among marine natural products, including among imidazoles/imidazolones [15].

**Scheme 6:**
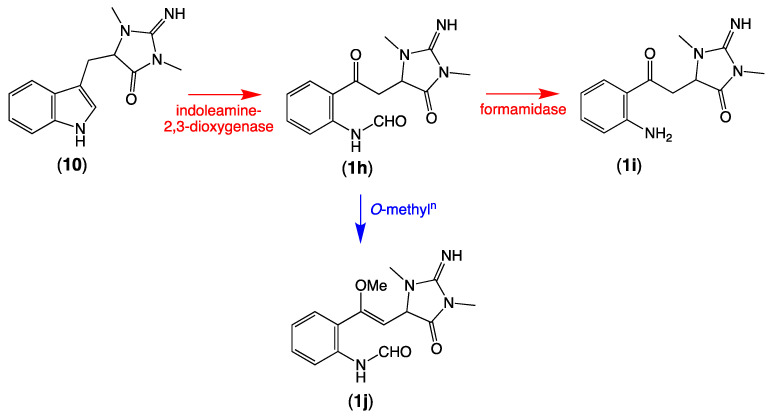
Possible biosynthetic link between 1′,8-dihydroaplysinopsin (**10**) and **1h**–**1j**.

**Scheme 7:**
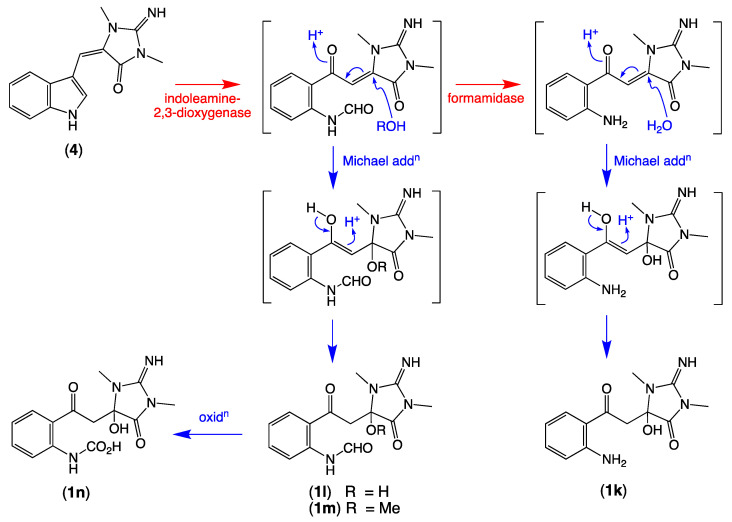
Possible biosynthetic link between aplysinopsin (**4**) and **1k**–**1n.**

Of note, the thorectandrin GNPS cluster also featured nodes tentatively attributed to debromo analogues (**1h**, M + H *m*/*z* 289, C_14_H_16_N_4_O_3_; **1i**, M + H *m*/*z* 261, C_13_H_16_N_4_O_2_) **1j**, M + H *m*/*z* 303, C_15_H_18_N_4_O_3_), consistent with indoleamine 2,3-dioxygenase-like enzyme transformation of 1′,8-dihydroaplysinopsin (**10**) (Scheme 6, Table 2, Figure 3). It also revealed nodes tentatively attributed to solvolysis adduct (**1k**, M + H *m*/*z* 277, C_13_H_16_N_4_O_3_; **1l**, M + H *m*/*z* 305; **1m**, M + H *m*/*z* 319, C_15_H_18_N_4_O_5_; **1n**, M + H *m*/*z* 321), derived from intermediates generated by indoleamine 2,3-dioxygenase-like transformation of aplysinopsin (**4**) (Scheme 7, Table 2, Figure 3).

## 3. Conclusions

Our application of the GNPS molecular networking to a library of Southern Australian marine sponges reinforced the value of this approach, in both dereplicating and prioritizing extracts for detailed investigation, and in guiding the discovery of new scaffolds. It also revealed itself to be a valuable tool for interrogating the halo of co-clustering minor analogues (including solvolysis artifacts), chemistry that typically defines traditional methods of isolation and structure elucidation.

Our study of an Australian deep water (45 m) marine sponge, *Thorectandra choanoides* (CMB-01889), lead to the discovery of a new class of alkaloid, thorectandrin A (**1**). In proposing a biosynthetic origin of **1**, we speculated that *Thorectandra choanoides* (CMB-01889) possesses enzymes functionally related to indoleamine 2,3-dioxygenase, leading to transformation of the known (albeit rare and minor) sponge natural product 6-bromo-1′,8-dihydroaplysinopsin (**2**) to its ring-opened *N*-formyl derivative **1a**, which was then rapidly hydrolyzed to thorectandrin A (**1**). Supportive of this hypothesis, we tentatively identified **1a** in the thorectandrin GNPS cluster, along with the O-methyl product **1b**, debromo analogues **1h**–**1j**, solvolysis adducts **1c**–**1g** and **1k**–**1m**, and a natural/artifact oxidation product **1n**. Studies into minor solvolysis artifacts led us to speculate that *Thorectandra choanoides* (CMB-01889) was capable of producing four known alkaloids, 6-bromo-1′,8-dihydroaplysinopsin (**2**) [7], 6-bromoaplysinopsin (**3**) [7], aplysinopsin (**4**) [16], and 1′,8-dihydroaplysinopsin (**10**) [7], all of which were susceptible to a sponge indoleamine 2,3-dioxygenase-like enzyme. Where the 1′,8-dihydro alkaloids **2** and **10** were fully transformed by this enzyme to stable ring-opened analogues **1** and **1a**–**1b**, and **1h**–**1j**, respectively, the related conjugated scaffolds **3** and **4** were fully transformed to highly reactive Michael acceptors that underwent complete transformation to **1c**–**1g**, and **1k**–**1n**, respectively.

As members of the aplysinopsin family of marine natural product are long known for their biological properties (i.e., anticancer, antibiotic, antidepressant, antimalarial, and antimicrobial properties) [11], the realisation that they are possible substrates for indoleamine 2,3-dioxygenases is significant. With human indoleamine 2,3-dioxygenase upregulated in key human tissues (i.e., small intestine and lung), and a number of cancers (i.e., acute myeloid leukemia, ovarian, and colorectal carcinoma), knowledge that aplysinopsins are substrates, and yield potent Michael acceptors, could inform future development of this pharmacophore. For example, one might take advantage of the tissue selective abundance of human indoleamine 2,3-dioxygenases for in situ production of highly reactive Michael acceptors (i.e., as warheads within cancer cells). Alternatively, one might seek to diminish the susceptibility of aplysinopsin chemotherapeutics to indoleamine 2,3-dioxygenases, to improve in vivo pharmacokinetics. Either way, an understanding of the biotransformation of aplysinopsins to thorectandrins and spiroreticulatine, and the Michael acceptor status of key intermediates, would inform researchers seeking to exploit the therapeutic potential of these closely related and uniquely marine pharmacophores.

## 4. Materials and Methods

### 4.1. General Experimental Procedures

Chiroptical measurements ([α]_D_) were obtained on a JASCO P-1010 polarimeter (JASCO International Co. Ltd., Tokyo, Japan) in a 100 × 2 mm cell at 23 °C. Electronic Circular Dichroism (ECD) measurement were obtained on a JASCO J-810 spectropolarimeter (JASCO International Co. Ltd., Tokyo, Japan) in a 0.1 cm path-length cell. Nuclear magnetic resonance (NMR) spectra were acquired on a Bruker Avance 600 MHz spectrometer (Bruker Pty. Ltd., Alexandria, Australia) with a 5 mm PASEL ^1^H/D-^13^C Z-Gradient probe at 25 °C in methanol-*d*_4_ by referencing to residual ^1^H or ^13^C signals (δ_H_ 3.30 and δ_C_ 49.15). High-resolution ESIMS spectra were obtained on a Bruker micrOTOF mass spectrometer (Bruker Daltonik Pty. Ltd., Preston, Australia) by direct injection in MeOH at 3 μL/min, using sodium formate clusters as an internal calibrant. Semi-preparative HPLC was performed using Agilent 1100 series HPLC instrument (Agilent Technologies Inc., Mulgrave, Australia) with corresponding detector, fraction collector and software inclusively. Analytical-grade solvents were used for extractions and partitions. Chromatography solvents were of HPLC grade and filtered/degassed through 0.45 μm polytetrafluoroethylene (PTFE) membrane prior to use. Deuterated solvents were purchased from Cambridge Isotopes (Cambridge Isotope Laboratories, Tewksbury, MA, USA). The human colorectal (SW620) and lung (NCI-H460) carcinoma cell lines were kindly provided by Susan E. Bates and Robert W. Robey of the National Cancer Institute, Bethesda, MD, USA.

### 4.2. Collection and Taxonomy

Sponge specimen CMB-01889 was collected in July 1995 using epibenthic sled (RV Franklin vessel) at a depth of 45 m in the Great Australian Bight. The specimen was immediately frozen and transported at 0 °C to the laboratory, where it was thawed, documented, diced, and stored in EtOH, at −30 °C prior to chemical investigation. The specimen was taxonomically classified as a *Thorectandra choanoides* (Class Demospongiae, Order Dictyoceratida, Family Thorectidae).

### 4.3. Extraction and Fractionation

An aliquot (60 mL) of the EtOH crude extract was decanted, concentrated in vacuo, and partitioned between *n*-BuOH (20 mL) and H_2_O (20 mL) (Figure S3). MS analysis of the partitions indicated localization of the target GNPS cluster in the *n*-BuOH partition. The *n*-BuOH soluble material was dissolved in MeOH and subjected to HPLC fractionation (Agilent Zorbax SB-CN 9.4 × 250 mm, 5 μm, 3 mL/min gradient elution over 25 min from 10% MeCN/H_2_O to 60% MeCN/H_2_O, with constant 0.01% TFA modifier), to yield thorectandrin A (**1**) (*t*_R_ = 15.2 min, 1.2 mg, 0.6%) (Figure S4).

### 4.4. Global Natural Product Social (GNPS) Molecular Networking

Aliquots (10 μL) of the *n*-BuOH soluble stock plates prepared from 980 Southern Australian marine sponges were dispensed into 96-well plates, dried under N_2_ gas, resuspended in DMSO (10 μL). A total of 0.1 μL of DMSO aliquot was injected into an Agilent 6545 QTOF LC/MS (Agilent Technologies Inc., Mulgrave, Australia) equipped with an Agilent 1290 infinity II UHPLC system, utilizing an Agilent SB-C_8_ 1.8 μm, 2.1 × 50 mm column, with a 0.5 mL/min, 4.5 min gradient elution from 90% H_2_O/MeCN to 100% MeCN, followed by isocratic elution with 100% MeCN for 1 min, with a constant isocratic 0.1% formic acid modifier. The UPLC-QTOF-(+)MS/MS (Agilent Technologies Inc., Mulgrave, Australia) data were acquired for all samples at a fixed collision energy of 40 eV, converted from Agilent MassHunter data files (.d) to mzXML file format, and transferred to the GNPS server (gnps.ucsd.edu) [17]. The full MS/MS data of the 980 sponge extracts could be accessed (accessed on 20 December 2020) from ftp://massive.ucsd.edu/MSV000086621/. Molecular networking was performed using the GNPS data analysis workflow using the spectral clustering algorithm, and a cosine score of 0.7 and a minimum of 6 matched peaks. The resulting spectral networks were visualized using Cytoscape version 3.5.1 (open source software, https://cytoscape.org (accessed on 20 December 2020)) [18], where nodes represented parent *m*/*z* and edge thickness corresponded to cosine scores, which showed a network featuring ~43,000 nodes, and many hundreds of clusters (Figure S1). Careful review of this GNPS data highlighted a promising cluster (Figure S2) with possible new compounds, associated uniquely with only one sponge specimen, CMB-01889.

### 4.5. Metabolite Characterization

*Thorectandrin A* (**1**): light yellow oil; ${\left[\alpha \right]}_{D}^{22.6}$ 0 (*c* 0.08, MeOH); NMR (methanol-*d*_4_), Table 1 and Figures S5–S10. HRESIMS *m/z* 339.0460/341.0454 [M + H]^+^ (calculated for C_13_H_16_^79^BrN_4_O_2_, 339.0451; C_13_H_16_^81^BrN_4_O_2_, 341.0431).

## Figures and Tables

**Table 1:** NMR (600 MHz) data for thorectandrin A (**1**) in methanol-*d*_4_.

Position	δ_C_	δ_H_, Mult. (*J* in Hz)	COSY	HMBC	ROESY
3	197.8				
3a	116.1				
4	133.9	7.67, d (8.7)	5	6, 7a, 3	8a, 8b
5	119.4	6.73, br d (8.7)	4,7	3a, 7	
6	131.1				
7	120.7	6.97, br s	5	3a, 5, 6	
7a	154.1				
8	38.0	a. 3.94, dd (19.0, 3.5)	8b, 1′	1′, 5′, 3	4
		b. 3.67, dd (19.0, 3.9)	8a, 1′	1′, 3	4
1′	60.8	4.56, dd (3.9, 3.5)	8a, 8b	5′, 3	2′N-CH_3_
2′N-CH_3_	30.3	3.09, s		3′, 1′	1′
3′	160.3				
4′N-CH_3_	26.8	3.26, s		3′, 5′	
5′	173.8				

**Table 2:** Molecular formulae of compounds within the thorectandrin GNPS cluster.

*m*/*z*^a^ (M + H)	Molecular Formula	ΔmDa ^b^	MF Difference with 1
Bromo natural products			
339 (**1**)	C_13_H_15_BrN_4_O_2_	0.05	
367 (**1a**)	C_14_H_15_BrN_4_O_3_	−1.99	CO
353 (**1b**)	C_14_H_17_BrN_4_O_2_	0.78	CH_2_
Bromo natural product solvolysis adducts			
397 (**1c**)	C_15_H_17_BrN_4_O_4_	−1.64	CO + CH_2_O
355 (**1d**)	C_13_H_15_BrN_4_O_3_	0.29	O
369 (**1e**)	C_14_H_17_BrN_4_O_3_	−1.03	CH_2_O
383 (**1f**)	C_15_H_19_BrN_4_O_3_	−0.04	C_2_H_4_O
411 (**1g**)	C_17_H_23_BrN_4_O_3_	−0.50	C_4_H_8_O
Debromo natural products			
289 (**1h**)	C_14_H_16_N_4_O_3_	0.71	−Br + H + CO
261 (**1i**)	C_13_H_16_N_4_O_2_	−0.57	−Br + H
303 (**1j**)	C_15_H_18_N_4_O_3_	1.2	−Br + H + CH_2_
Debromo natural product solvolysis adducts			
277 (**1k**)	C_13_H_16_N_4_O_3_	−1.25	−Br + H + O
319 (**1m**)	C_15_H_18_N_4_O_4_	1.1	−Br + H + CO + OCH_2_

^a^*m*/*z* from GNPS nodes; ^b^ Difference between calculated and experimental *m*/*z* the latter measured individually from UPLC-QTOF data; NOTE: **1l** (M + H, *m*/*z* 305) and **1n** (M + H, *m*/*z* 321) are minor metabolites that precluded unambiguous measurement of accurate *m*/*z* and MF.

## Data Availability

The full MS/MS data of the 980 sponge extracts could be accessed from ftp://massive.ucsd.edu/MSV000086621/.

## Supplementary Materials

The following are available online at https://www.mdpi.com/1660-3397/19/2/97/s1. Full GNPS molecular network, isolation scheme and HPLC chromatogram, 1D and 2D NMR spectra, and HRMS spectrum and bioassay protocols.
