# Supplementary material for: New from Old: Thorectandrin Alkaloids in a Southern Australian Marine Sponge, Thorectandra choanoides (CMB-01889)
Source: Mar Drugs. 2021 Feb 9;19(2):97. doi: 10.3390/md19020097 (PMC7914882; doi:10.3390/md19020097)
Supplement: Supplementary file 1 [file marinedrugs-19-00097-s001.pdf]

Supporting Information

New from old: Thorectandrin alkaloids in a southern Australian marine sponge, *Thorectandra choanoides* (CMB-01889)

Shamsunnahar Khushi, Angela A. Salim, Ahmed H. Elbanna, Laizuman Nahar, Robert J. Capon*

Institute for Molecular Bioscience, The University of Queensland, St. Lucia, QLD 4072, Australia

*Correspondence: r.capon@uq.edu.au

List of Figures

Figure S1. A portion of the GNPS molecular networks for 960 marine n-BuOH extracts and 95 authentic standards from different classes of marine natural compounds. 3
Figure S2. GNPS molecular cluster uniquely associated with *Thorectandra choanoides* (CMB-01889).. 4
Figure S3. Fractionation scheme for *Thorectandra choanoides* (CMB-01889)...................... 5
Figure S4. Analytical HPLC chromatogram of CMB-01889 n-BuOH soluble fraction (inset-UV spectrum of **1**).. 5
Figure S5.^{1}H NMR (600 MHz, methanol-d_4) spectrum of thorectandrin A (**1**)...................... 6
Figure S6. ^{13}C NMR (150 MHz, methanol-d_4) spectrum of thorectandrin A (**1**)..................... 6
Figure S7. HSQC (600 MHz, methanol-d_4) spectrum of thorectandrin A (**1**).......................... 7
Figure S8. HMBC (600 MHz, methanol-d_4) spectrum of thorectandrin A (**1**)........................ 7
Figure S9. COSY (600 MHz, methanol-d_4) spectrum of thorectandrin A (**1**).......................... 8
Figure S10. ROESY (600 MHz, methanol-d_4) spectrum of thorectandrin A (**1**) 8
Figure S11. HRESIMS data for thorectandrin A (**1**) 9
Figure S12. ECD (top) and UV (bottom) spectra of thorectandrin A (**1**)……………………9
Figure S13. Antibacterial and antifungal assay results for thorectandrin A (**1**)……………..11
Figure S14. Cytotoxicity assay results for thorectandrin A (**1**)……………………………...11

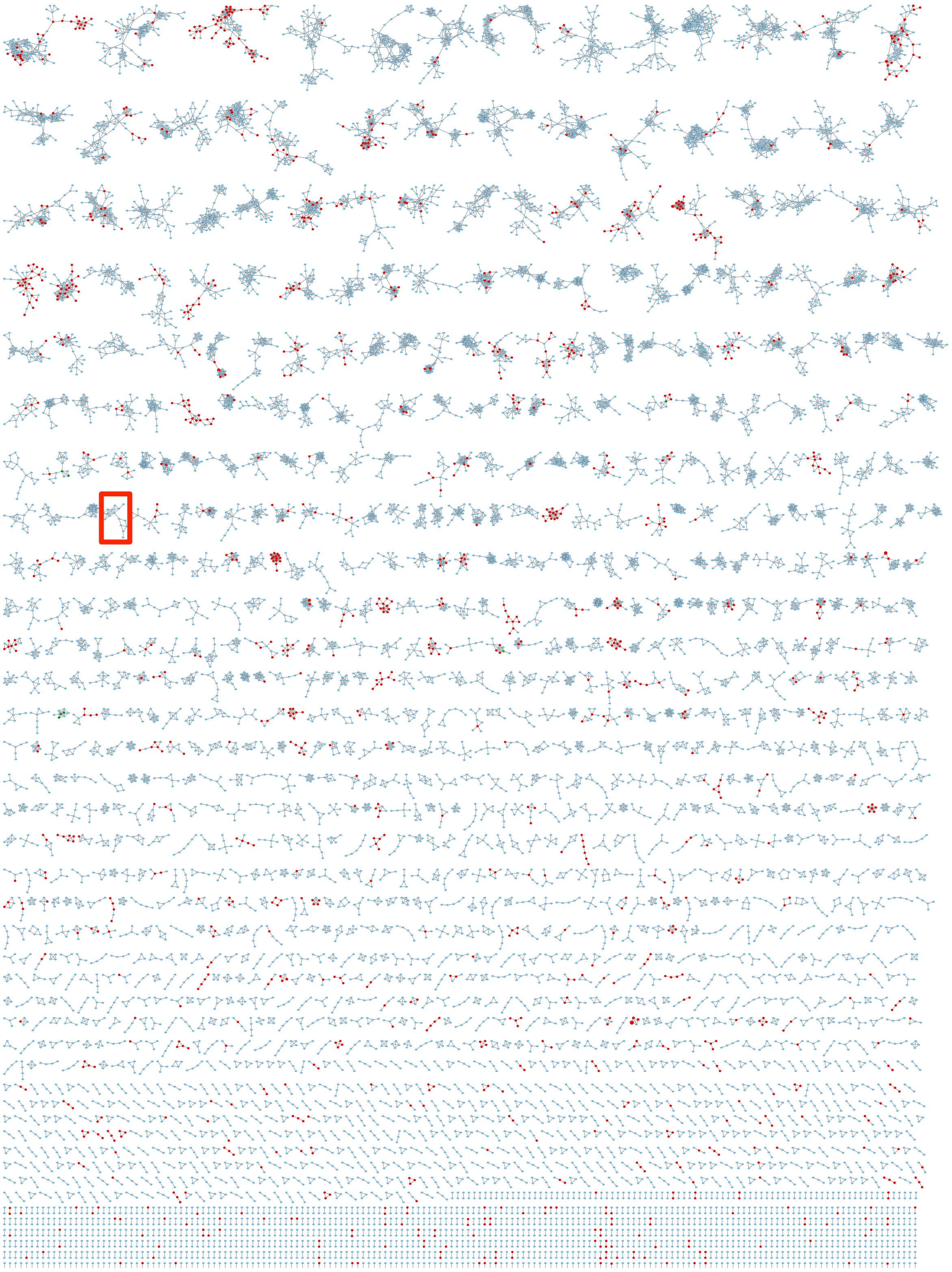

Figure S1. A portion of the GNPS molecular networks for 960 marine *n*-BuOH extracts and 95 authentic standards from different classes of marine natural compounds (red nodes represent known compounds). Red box: thorectandrins cluster (see Figure 2 for expansion).

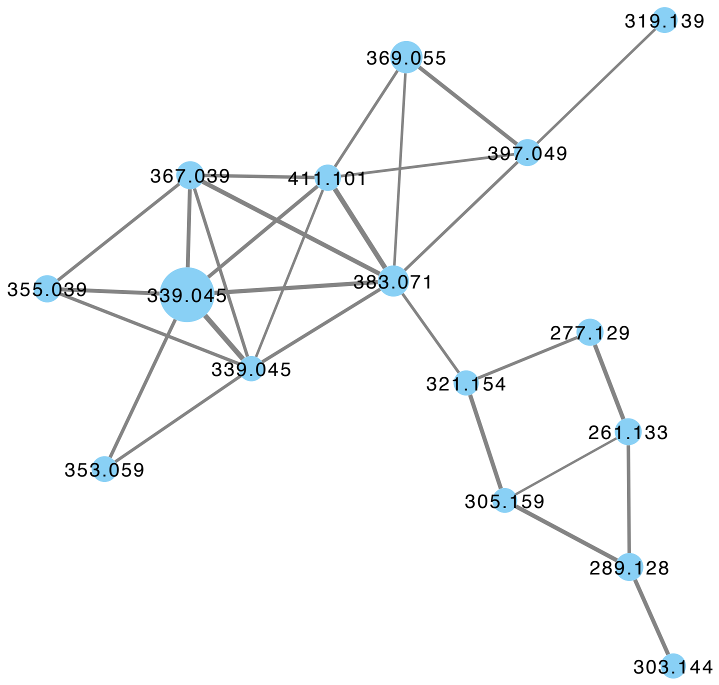

Figure S2. GNPS molecular cluster uniquely associated with *Thorectandra choanoides* (CMB-01889). Numbers on nodes correspond to m/z values.

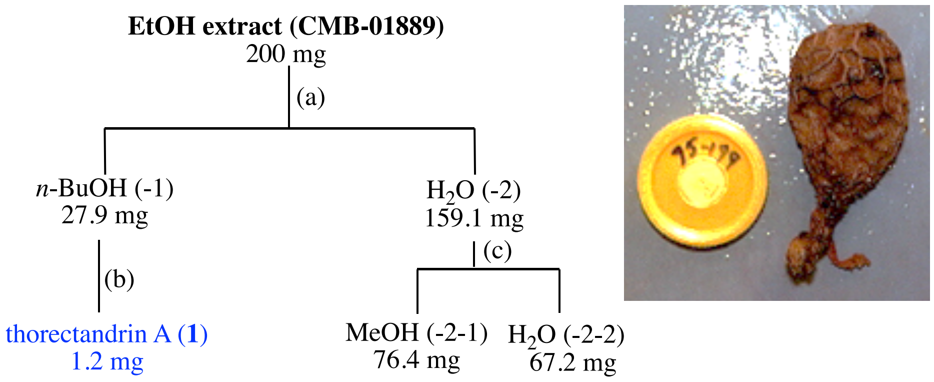

(a) Partition (*n*-BuOH and H_2O)
(b) Semi preparative HPLC (Agilent Zorbax SB-CN 9.4 mm × 25 cm, 5 μm, 3 mL/min)
(c) Trituration (MeOH and H_2O)

Figure S3. Fractionation scheme for *Thorectandra choanoides* (CMB-01889), inset: sponge picture.

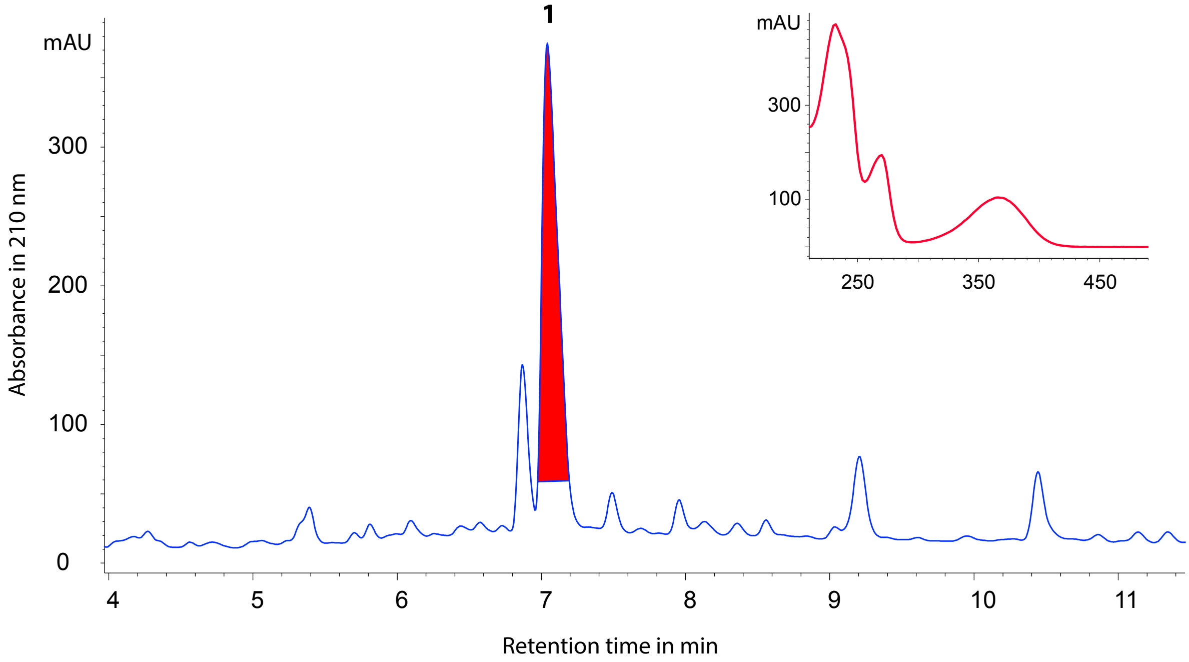

Figure S4. Analytical HPLC chromatogram of CMB-01889 n-BuOH soluble fraction (inset- UV spectrum of **1**)

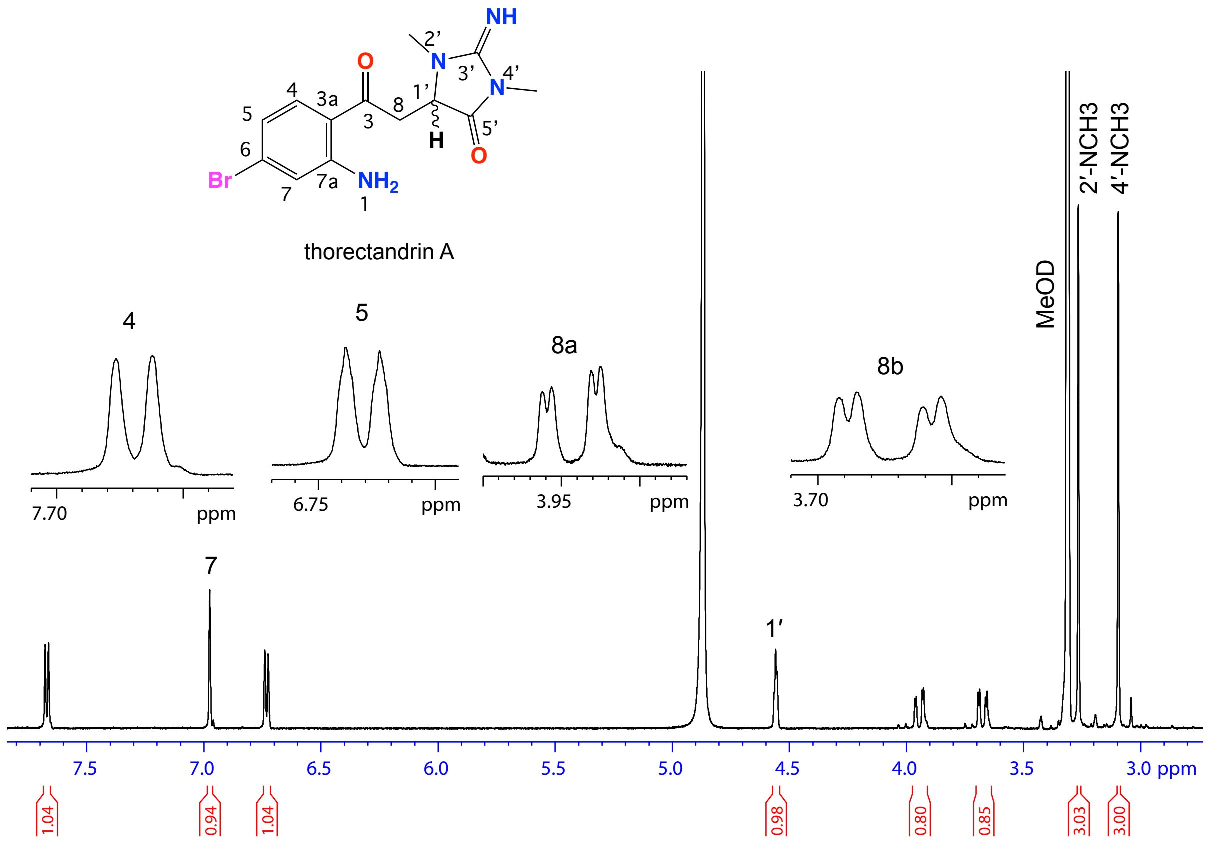

Figure S5.^{1}H NMR (600 MHz, methanol-d_4) spectrum of thorectandrin A (**1**)

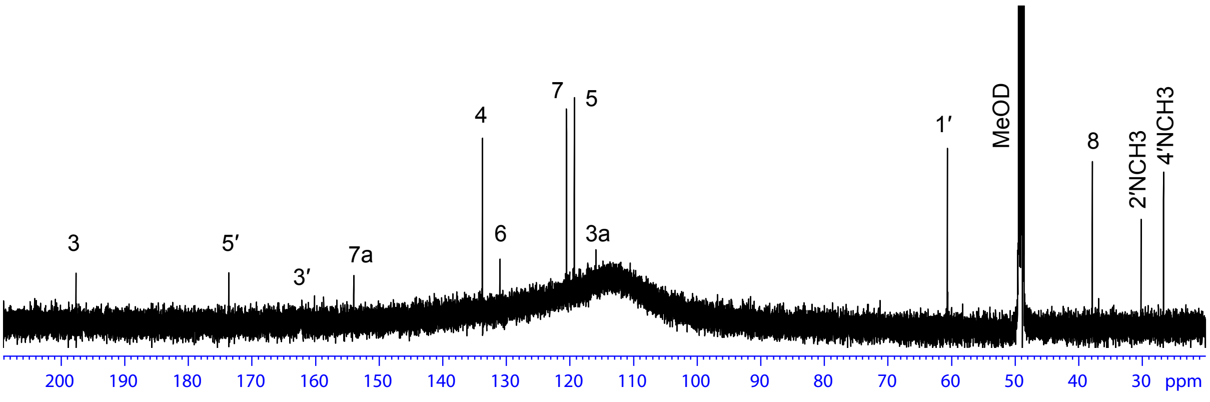

Figure S6. ^{13}C NMR (150 MHz, methanol-d_4) spectrum of thorectandrin A (**1**)

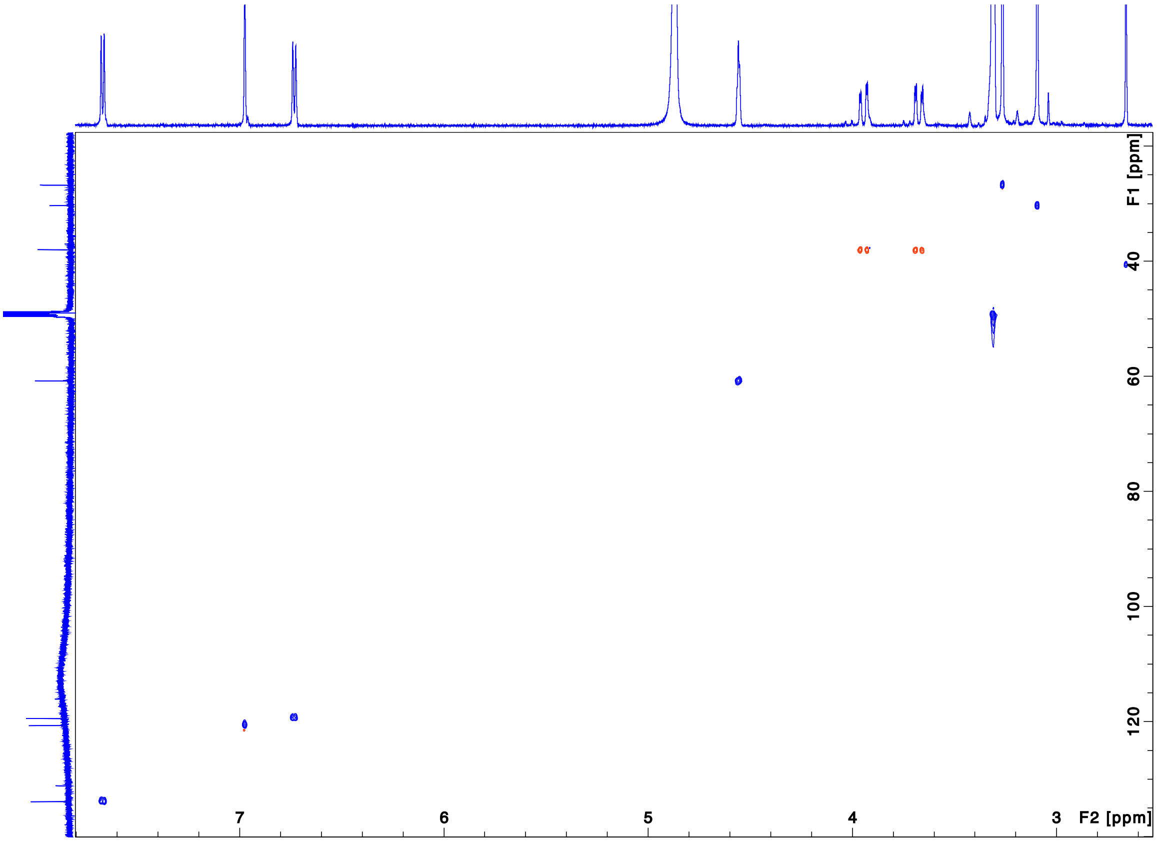

Figure S7. HSQC (600 MHz, methanol-d_4) spectrum of thorectandrin A (**1**)

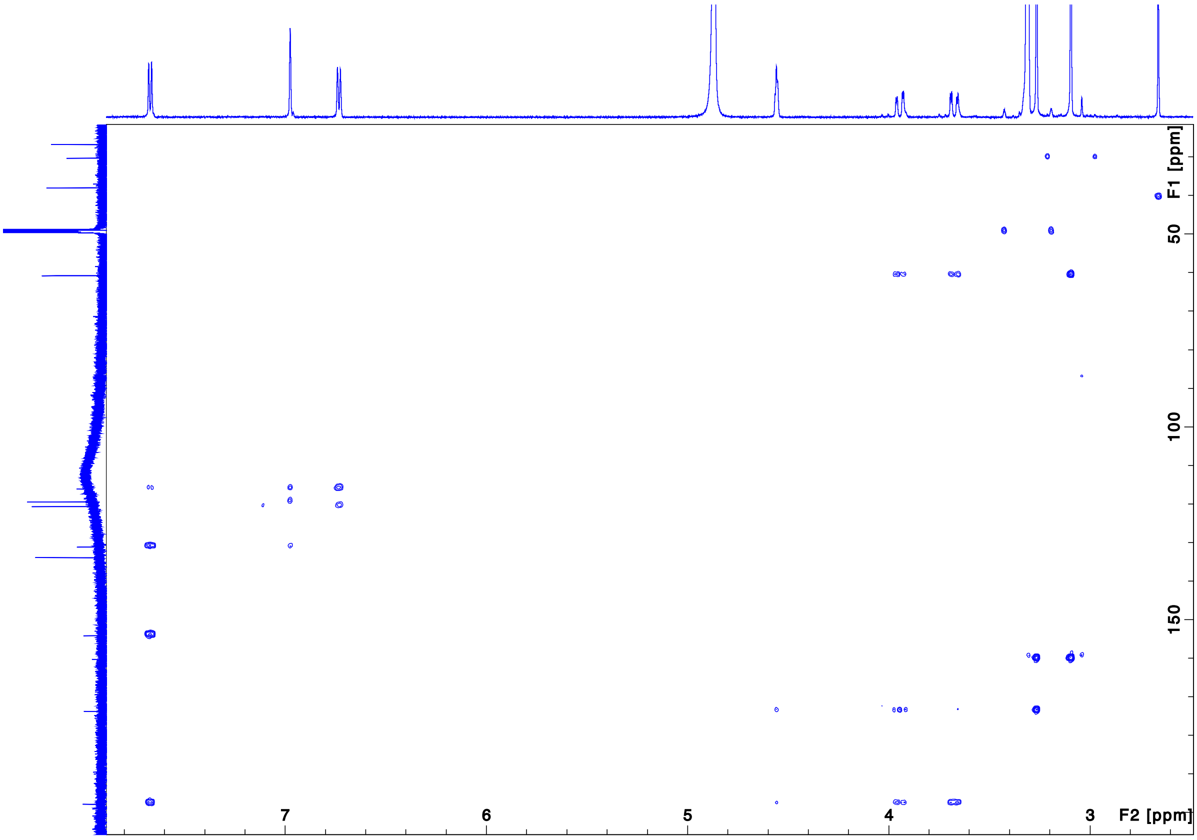

Figure S8. HMBC (600 MHz, methanol-d_4) spectrum of thorectandrin A (**1**)

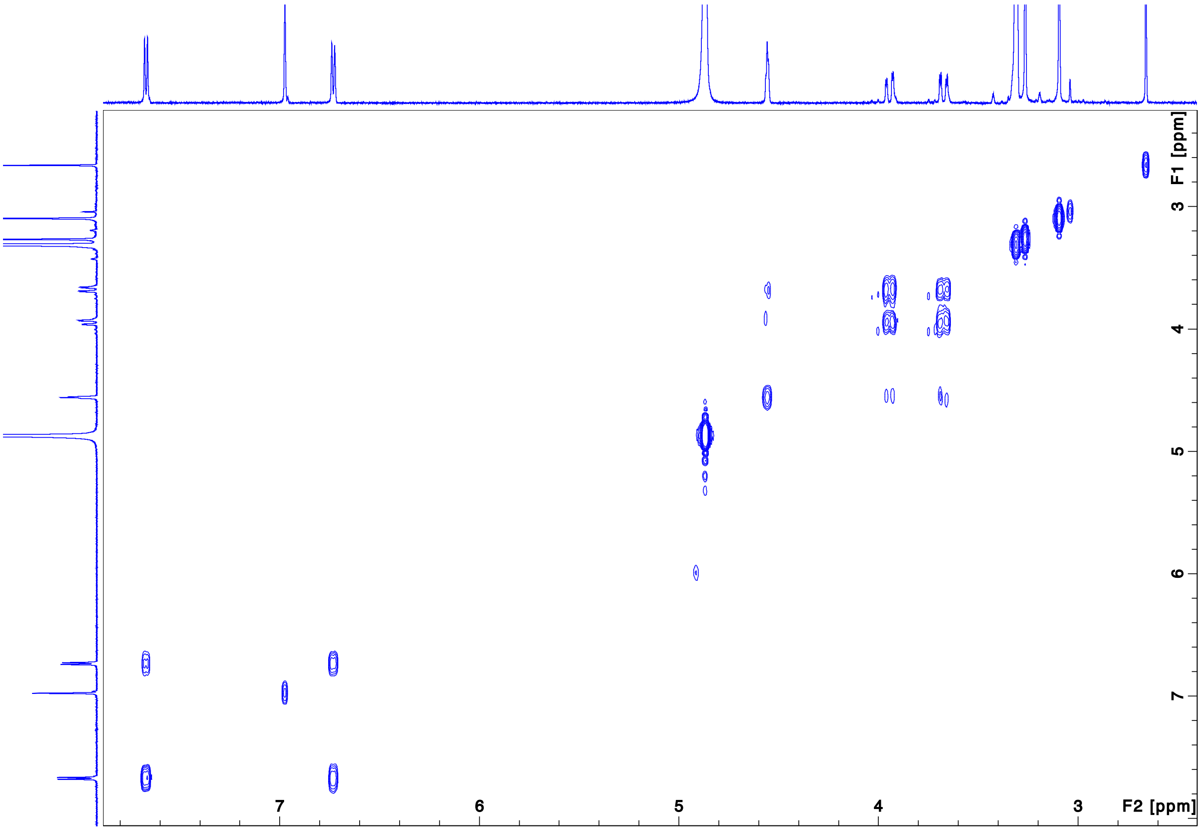

Figure S9.COSY (600 MHz, methanol-d_4) spectrum of thorectandrin A (**1**)

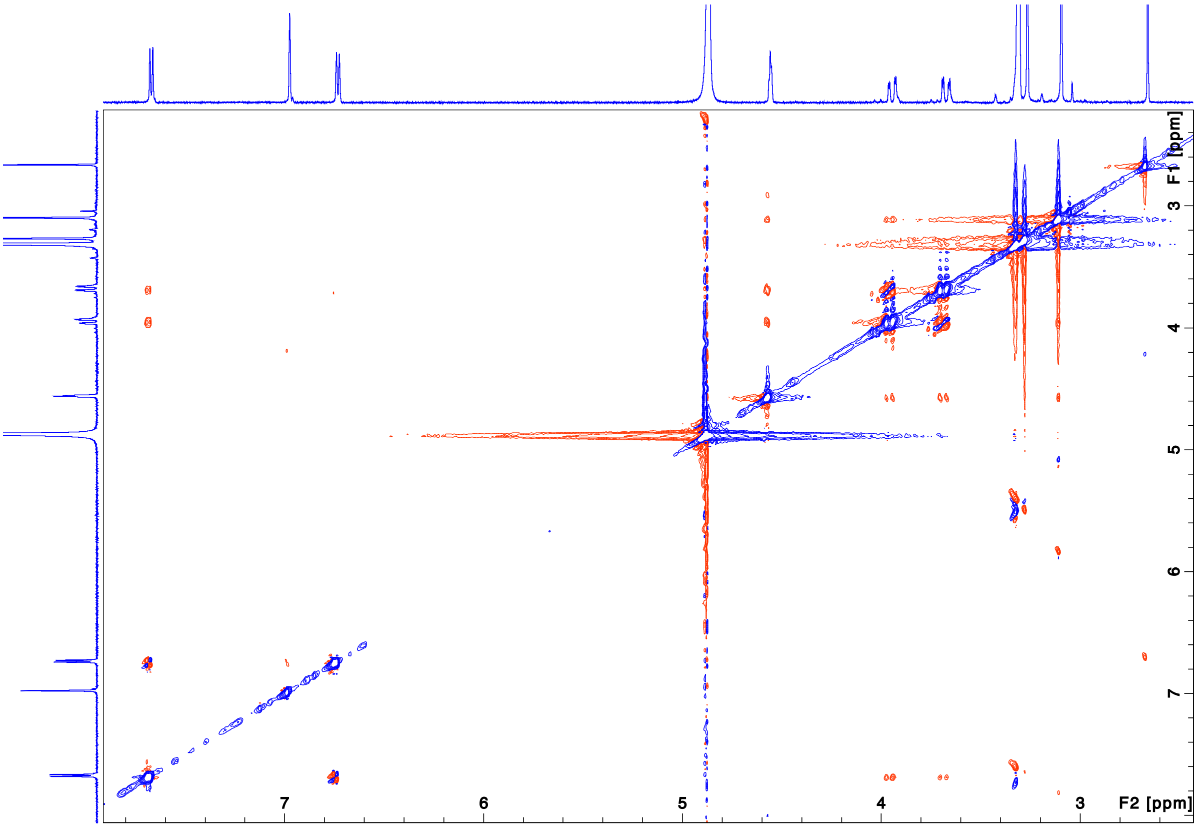

Figure S10.ROESY (600 MHz, methanol-d_4) spectrum of thorectandrin A (**1**)

Mass Spectrum Molecular Formula Report

Analysis Info

Analysis Name	D:\Data\s.khushi\cmb-01889-1-11-14.d	Acquisition Date	5/30/2019 12:20:38 PM
Method	tune-med_AP.m	Operator	a.salim
Sample Name		Instrument / Ser#	micrOTOF 213750.00232
Comment			

Acquisition Parameter

Source Type	ESI	Ion Polarity	Positive	Set Nebulizer	0.8 Bar
Focus	Not active			Set Dry Heater	180 °C
Scan Begin	100 m/z	Set Capillary	4500 V	Set Dry Gas	5.0 l/min
Scan End	1000 m/z	Set End Plate Offset	-500 V	Set Divert Valve	Source

Generate Molecular Formula Parameter

Formula, min.		
Formula, max.		
Measured m/z	Tolerance	Charge
Check Valence	Minimum	Maximum
Nirogen Rule	Electron Configuration	
Filter H/C Ratio	Minimum	Maximum
Estimate Carbon		

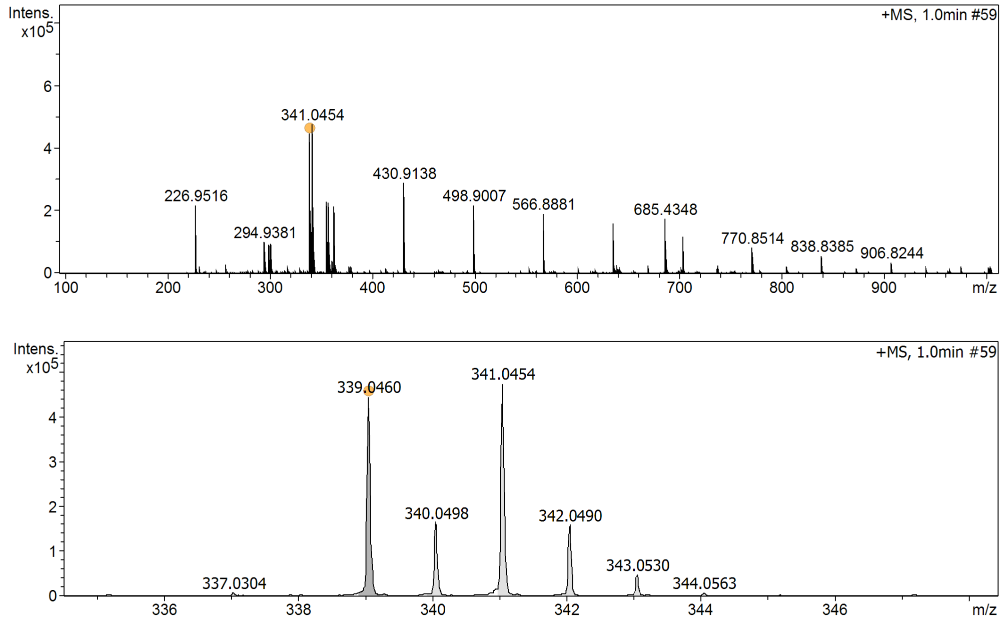

Meas. m/z	#	Ion Formula	m/z	err [ppm]	mSigma	# Sigma	Score	rdb	e⁻ Conf	N-Rule
339.0460	1	C13H16BrN4O2	339.0451	2.6	124.9	1	100.00	7.5	even	ok

Figure S11. HRESIMS data for thorectandrin A (**1**)

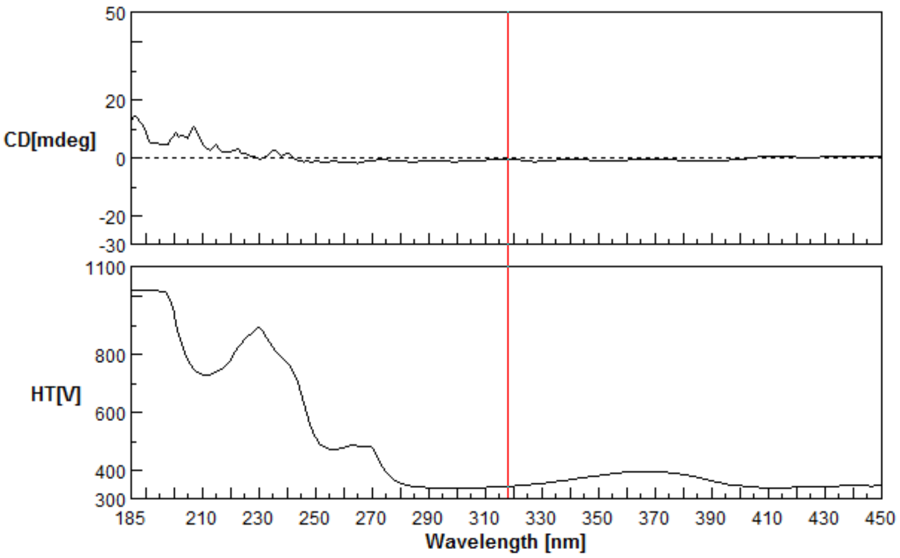

Figure S12. ECD (top) and UV (bottom) spectra of thorectandrin A (**1**)

Antibacterial assay

The bacterium to be tested was streaked onto a tryptic soy agar plate and incubated at 37 °C for 24 h. One colony was then transferred to fresh tryptic soy broth (15 mL) and the cell density adjusted to 5-6×10^5 CFU/mL. The compounds to be tested were dissolved in DMSO and diluted with H_2O to return 600 µM stock solutions (20% DMSO). The stock solutions were serially diluted to give concentration range of 600 µM to 0.2 µM in 20% DMSO/H_2O. An aliquot (10 µL) of each dilution was transferred to a 96-well microtiter plate and freshly prepared microbial broth (190 µL) was added to each well. The plates were incubated at 37 °C for 24 h and the optical density of each well was measured spectrophotometrically at 600 nm using POLARstar Omega plate (BMG LABTECH, Offenburg, Germany). Each test compound was screened against the Gram-negative bacterium *Escherichia coli* (ATCC 11775) and the Gram-positive bacterium *Bacillus subtilis* (ATCC 6051). Rifampicin was used as a positive control (1.2 µM, 40 µg/mL in 10% DMSO). The IC_{50} value was calculated as the concentration of the compound or antibiotic required for 50% inhibition of the bacterial cells using Prism 7.0 from GraphPad Software Inc. (La Jolla, CA). All experiments were performed in duplicate.

Antifungal assay

The fungus to be tested was streaked onto a sabouraud agar plate and incubated at 37 °C for 24 h. One colony was then transferred to fresh sabouraud broth (15 mL) and the cell density adjusted to 5-6×10^5 CFU/mL. The compounds to be tested were dissolved in DMSO and diluted with H_2O to return 600 µM stock solutions (20% DMSO). The stock solutions were serially diluted to give concentration range of 600 µM to 0.2 µM in 20% DMSO/H_2O. An aliquot (10 µL) of each dilution was transferred to a 96-well microtiter plate and freshly prepared microbial broth (190 µL) was added to each well to give a final concentration of 30 µM to 0.01 µM per well. The plates were incubated at 37 °C for 24 h and the optical density of each well was measured spectrophotometrically at 600 nm using POLARstar Omega plate (BMG LABTECH, Offenburg, Germany). Each test compound was screened against *Candida albicans* (ATCC 10231). Nystatin was used as a positive control (1.1 µM, 40 µg/mL in 10% DMSO). The IC_{50} value was calculated as the concentration of the compound or antibiotic required for 50% inhibition of the bacterial cells using Prism 7.0 from GraphPad Software Inc. (La Jolla, CA). All experiments were performed in duplicate.

Cytotoxicity assay

Adherent human colorectal (SW620) and lung (NCI-H460) carcinoma cells were cultured in RPMI medium 1640. All cells were cultured as adherent mono layers in flasks supplemented with 10% foetal bovine serum, L-glutamine (2 mM), penicillin (100 unit/mL) and streptomycin (100 µg/mL), in a humidified 37 °C incubator supplied with 5% CO_2. Briefly, cells were harvested with trypsin and dispensed into 96-well microtiter assay plates at 2,000-5,000 cells/well after which they were incubated for 18 h at 37 °C with 5% CO_2 (to allow cells to attach as adherent mono layers). Test compounds were dissolved in 20% DMSO in PBS (v/v) and aliquots (10 µL) applied to cells over a series of final concentrations ranging from 10 nM to 30 µM. After 48 h incubation at 37 °C with 5% CO_2 an aliquot (20 µL) of MTT in PBS (5 mg/mL) was added to each well (final concentration 0.5 mg/mL), and microtiter plates were incubated for a further 4 h at 37 °C with 5% CO2. After final incubation, the medium was aspirated, and precipitated formazan crystals dissolved in DMSO (100 µL/well). The absorbance of each well was measured at 580 nm with a PowerWave XS Microplate Reader from Bio-

Tek Instruments Inc. (Winooski, VT). IC_{50} values were calculated using Prism 7.0 (GraphPad Software Inc., La Jolla, CA), as the concentration of analyte required for 50% inhibition of cancer cell growth (compared to negative controls). Negative control comprised of 1% aqueous DMSO, while positive control was doxorubicin. All experiments were performed in duplicate.

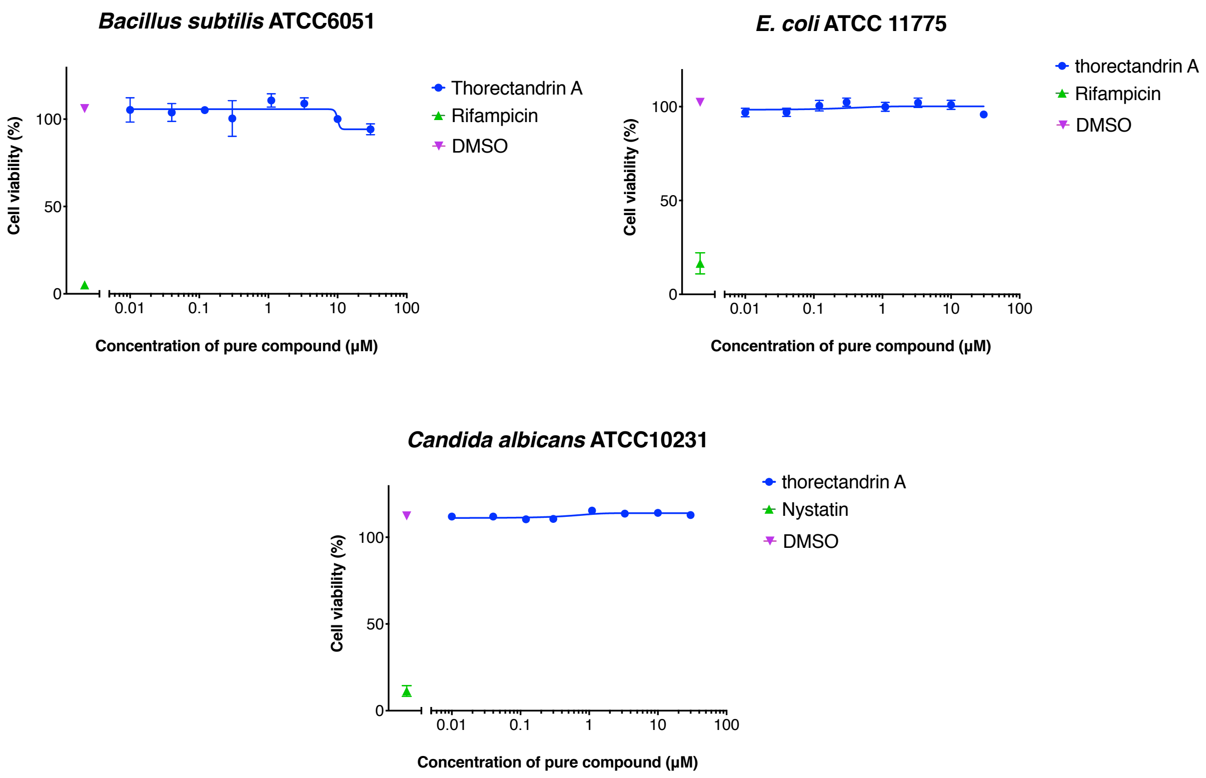

Figure S13. Antibacterial and antifungal assay results for thorectandrin A (**1**)

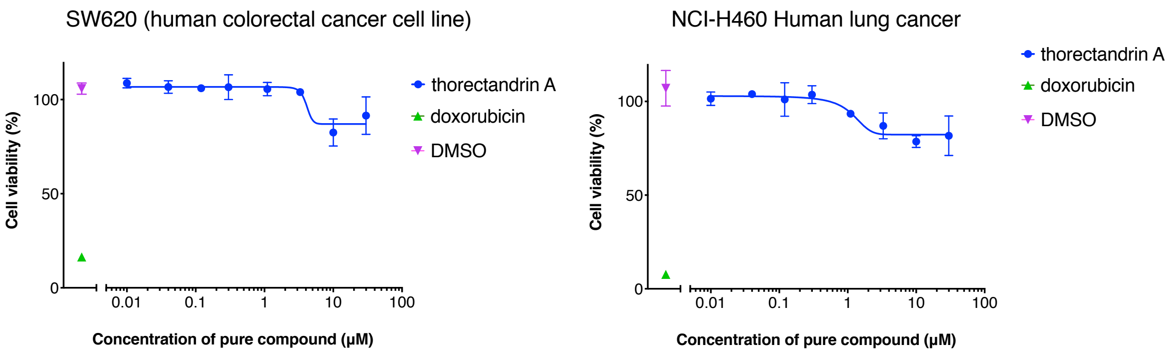

Figure S14. Cytotoxicity assay result for thorectandrin A (1)
